# Multifrequency Microwave Radiometry for Characterizing the Internal Temperature of Biological Tissues

**DOI:** 10.3390/bios13010025

**Published:** 2022-12-26

**Authors:** Enrique Villa, Beatriz Aja, Luisa de la Fuente, Eduardo Artal, Natalia Arteaga-Marrero, Gara Ramos, Juan Ruiz-Alzola

**Affiliations:** 1 Grupo Tecnología Médica IACTEC, Instituto de Astrofísica de Canarias (IAC), 38205 San Cristóbal de La Laguna, Spain; 2 Departamento de Ingeniería de Comunicaciones, Universidad de Cantabria, Plaza de la Ciencia s/n, 39005 Santander, Spain; 3 Instituto Universitario de Investigaciones Biomédicas y Sanitarias (IUIBS), Universidad de Las Palmas de Gran Canaria, 35016 Las Palmas de Gran Canaria, Spain; 4 Departamento de Señales y Comunicaciones, Universidad de Las Palmas de Gran Canaria, 35016 Las Palmas de Gran Canaria, Spain

**Keywords:** microwave radiometry, multifrequency, pseudo-correlation receiver, thermometry, temperature retrieval

## Abstract

The analysis of near-field radiometry is described for characterizing the internal temperature of biological tissues, for which a system based on multifrequency pseudo-correlation-type radiometers is proposed. The approach consists of a new topology with multiple output devices that enables real-time calibration and performance assessment, recalibrating the receiver through simultaneous measurable outputs. Experimental characterization of the prototypes includes a well-defined calibration procedure, which is described and demonstrated, as well as DC conversion from the microwave input power. Regarding performance, high sensitivity is provided in all the bands with noise temperatures around 100 K, reducing the impact of the receiver on the measurements and improving its sensitivity. Calibrated temperature retrievals exhibit outstanding results for several noise sources, for which temperature deviations are lower than 0.1% with regard to the expected temperature. Furthermore, a temperature recovery test for biological tissues, such as a human forearm, provides temperature values on the order of 310 K. In summary, the radiometers design, calibration method and temperature retrieval demonstrated significant results in all bands, validating their use for biomedical applications.

## 1. Introduction

The characterization of the natural electromagnetic (EM) radiation emitted from a body is a key aspect in many areas of interest, such as radio astronomy [1], security systems [2] or medicine [3]. The analysis of the regions of the EM spectrum, spanning from a few Hz up to 10${}^{25}$ Hz, provides appreciable developments in communication systems, fundamental research or medical applications.

Specifically, medical technology involves the use of diverse techniques to improve diagnosis or treatment procedures. Among these techniques, those focused on tracking body temperatures are of special concern since they enable medical practitioners to analyze the difference between external and internal temperatures. Research activity has demonstrated that the core body and skin temperatures differ significantly, reaching up to ±2 °C for healthy people [4,5,6]. Herein lies the importance of characterizing core temperature variations since deviations may be a clear indicator of body dysfunction, and these fluctuations may be detected prior to the appearance of anomalies at the skin surface.

External measurements of body tissue’s temperatures in clinical applications include diverse techniques, such as magnetic resonance imaging (MRI), X-rays, computer tomography (CT), ultrasound and infrared thermography. MRI offers high spatial resolution but lacks portability [7]. X-rays, as ionizing radiation, can be harmful to biological tissues [4]. CT and ultrasound use external applicators to obtain the measurements. In addition, their cost, sensitivity or discomfort, depending on the modality, are common limitations of these techniques [8]. Infrared thermography constitutes a non-contact, non-invasive and fast approach to measuring temperature [9,10]. However, the measurements provided are confined to the superficial tissues. On the other hand, internal measurements are usually performed employing invasive techniques, commonly through oral, rectal or esophageal access, causing discomfort to patients [11,12]. These techniques are not optimal for long-term monitoring since they can cause irritation and are difficult to employ [4]. The non-fixed location of the sensor can produce gradients that increase the uncertainty of the measurement [13] and, even more, they are affected by simultaneous occurrences between external liquids and the probe within the body, increasing the measurement error [14].

**Figure 1:**
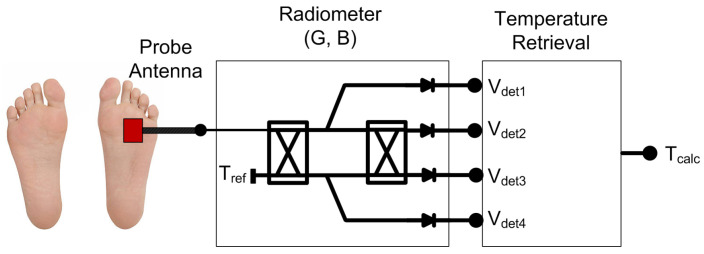
Illustration of a MWR system (G = gain; B = bandwidth) for estimating internal temperature, using a probe antenna located on the superficial skin and the subsequent receiver to detect and retrieve the temperature.

Hence, the main goal is to provide a non-ionizing, non-invasive, inexpensive, fast and passive system that is able to penetrate into the tissues up to centimeters [15,16]. Microwave radiometry (MWR) is an inherently safe technique to characterize internal temperatures by measuring variations of the natural thermal electromagnetic radiation emitted from a body or object. The system is basically composed of a probe antenna followed by a sensitive receiver, as shown in Figure 1.

Recently, there has been growing interest in MWR, and significant results were demonstrated within the biomedical field, focusing on anomalies in breast, brain, carotid artery, back pain or diabetic foot, among others [17,18]. Furthermore, MWR can complement superficial temperature measurements provided by infrared sensors, in cases in which a high risk of the appearance of skin ulcers is present, such as diabetic foot patients [19,20]. In fact, MWR has even been employed for the diagnosis of COVID-19 disease [21]. In addition, multifrequency MWR has demonstrated great potential in the detection of tumors aided by phantoms [22], the diagnosis of breast carcinoma [23], monitoring of brain temperatures in infants [24,25] and functional diagnostics of the brain [26].

New radiometer approaches have attempted to reduce the impact of the receiver in the measured temperature in terms of gain instabilities, system temperature and reflection variations in the interface between the antenna and the human body [27,28,29,30]. The work presented in this paper is aimed at the development of a multifrequency response microwave system for biomedical applications, which retrieves an unknown temperature of the biological tissue or object under investigation. The multifrequency radiometer is based on a pseudo-correlation configuration [31] that enables real-time calibration to correct receiver drifts. The pseudo-correlation configuration is proposed to reduce the dependence on gain fluctuations, whereas higher stability and observation time are also achieved compared to The Dicke solutions [32,33]. The performance is based on a multiple-output system that correlates two input signals to either measure each one at a single output or the combination of both of them at two additional outputs. The set of output voltages enables the recalibration of the receiver, correcting its drifts to perform temperature retrieval. The system noise temperature of a receiver is degraded by the input switch in Dicke topologies [27,28,29,30]. Consequently, the proposed topology includes low-noise amplifiers located just after the antenna to minimize the impact of the receiver. The work presented here extends the simulation results previously described [31] to calibrated sensors, including DC conversion through a multifrequency system. Zero-bias square-law detectors are employed to convert the microwave input power to output voltages. The temperature recovery provided by the radiometers is validated using representative broadband noise sources, as well as biological tissues (human forearm). As a multifrequency radiometric receiver, a set of frequencies is selected to reach different penetration depths within tissues, so the center frequencies are optimized to reach around 20 mm penetration depth. The full characterization of the radiometers and their temperature retrieval are described.

## 2. Multifrequency Pseudo-Correlation Radiometer

### 2.1. Operation Frequencies

Temperature patterns of subcutaneous tissues can be obtained by employing MWR systems [16], providing in-depth measurements of centimeters, roughly on the order of 5 cm at 0.5 GHz to 1 cm at 5 GHz [34,35]. These measurements are particularly significant when low frequencies are considered since below 6 GHz biological tissues are almost transparent to microwave radiation, and internal distances similar to the operating wavelength can be considered [15].

This work focuses on the detection of subcutaneous temperature anomalies for medical applications, particularly for biological tissues in which an adipose layer is not present, such as carotid artery diseases [36] or diabetic foot neuropathy [20]. As an initial approach, a single muscle layer is considered, since the skin layer is significantly thinner in comparison, to estimate the penetration depths and obtain the operating frequencies of the set of radiometers. Alternatively, a stack of layers composed of skin and muscle tissues could be considered instead.

**Figure 2:**
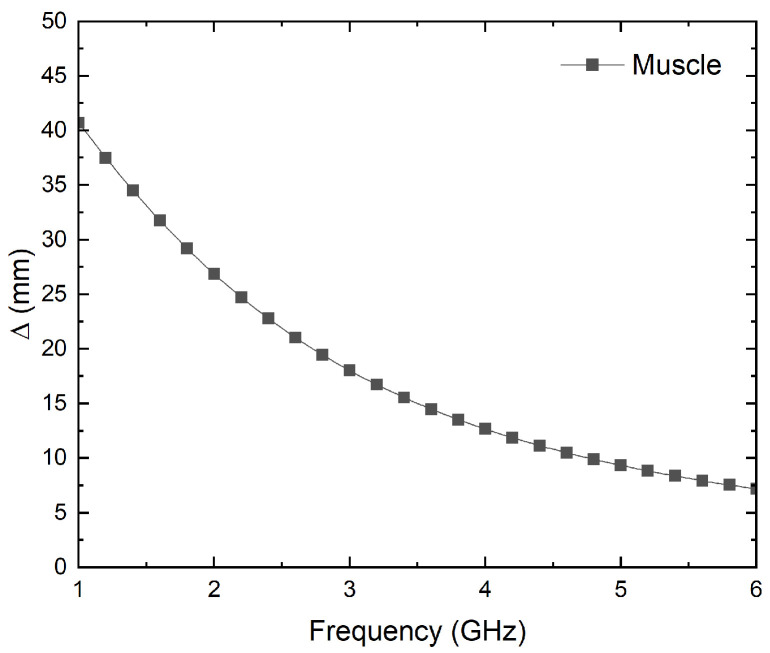
Simulation of the penetration depth versus frequency for muscle tissue [37].

The penetration depth, Δ, depends on the frequency of the electromagnetic field, as well as on the characteristics of the material or tissue under consideration. For low frequencies, the permittivity of the material is relatively high, and thus, the conductivity is low. As a result, the electromagnetic wave can propagate through the tissues without too much attenuation. At higher frequencies, the losses in the material are increased, and hence the penetration depth decreases [34]. The penetration depth for lossy materials is given by [35]

(1) $$\begin{array}{c} \Delta =\frac{\sqrt{2}\cdot c}{2\cdot \pi \cdot f}\cdot \frac{1}{\sqrt{\varepsilon_{r}^{\prime }\cdot \mu_{r}^{\prime }}\cdot \sqrt{\sqrt{1+\tan^{2}\delta }-1}} \end{array}$$

where *c* is the speed of light in vacuum, *f* is the frequency, ε_r_′ is the relative permittivity of the material, μ_r_′ is the relative permeability of the material, and tan δ is the loss tangent of the material. As stated before, muscle tissue [37] is assumed to approximate the penetration depth. Figure 2 depicts the depth calculated versus frequency up to 6 GHz. As can be seen, in the frequency range from 2.5 to 4.5 GHz, depth values between 20 and 10 mm are reached.

The operation frequencies should provide a reasonable compromise between spatial resolution, in terms of the size, and in-depth measurements in lossy tissues to achieve accurate results [28]. The frequency band should be as quiet as possible with low electromagnetic interference [38]. Therefore, three different frequency bands are proposed and listed in Table 1, which allow depth measurements of up to approximately 20 mm.

### 2.2. Receiver Design

**Figure 3:**
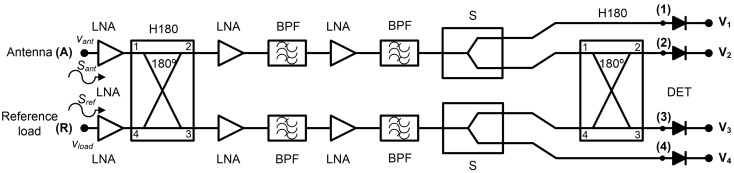
Proposed pseudo-correlation schematic using 180° hybrid couplers to correlate the signals and Wilkinson power dividers.

The receiver topology is based on a pseudo-correlation scheme [31]. This configuration allows the simultaneous measurement of a set of output signals, which are proportional to the input ones and their combination. The experimental work described includes the conversion from microwave power to DC voltage using square-law detectors. The configuration of the proposed multifrequency radiometers is shown in Figure 3. New 180° hybrid couplers are custom-designed, and detection stages are included, whose contributions are also added to the calibration procedure. These items are further described below. The cascaded double-section filtering and amplification stage is employed in between the hybrid couplers to strictly confine the band, reducing undesired noise power entering the receivers. Additionally, the power level is set into the detectable window of the detectors with further amplification. The analysis of this configuration, without considering the detectors, indicates that the output signal at label (2) is proportional to the antenna input, this is the detected voltage V_2_ ∝ S_ant_, whereas the output signal (3) is then proportional to the reference load, so the detected voltage is V_3_ ∝ S_ref_.

**Figure 4:**
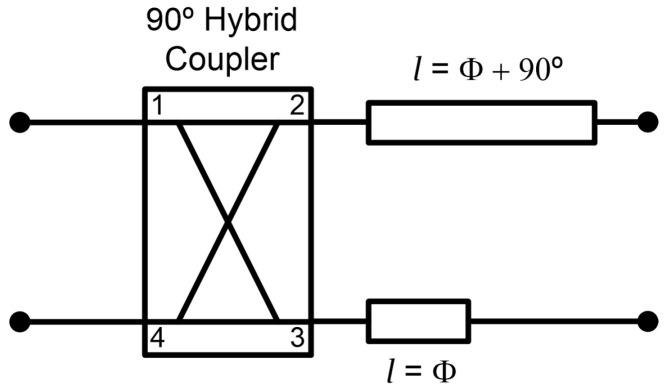
Configuration of the 180° hybrid coupler by means of a 90° hybrid coupler and a 90° phase shifter centered at each frequency band.

The radiometers are partially designed using commercial-off-the-shelf (COTS) components, listed in Table 2. In addition, custom grounded coplanar waveguide (CPWG) 90° differential phase shifters are designed for each frequency band (B1, B2 and B3) in order to accomplish a 180° hybrid coupler together with a 90° hybrid coupler. They are composed of a quarter wavelength transmission line related to a straight line, which differs 90° in phase at the center frequency of each band. Each transmission line is connected to the outputs of the 90° hybrid coupler, as shown in Figure 4. The 90° phase shifters are designed on CLTE-XT substrate (0.254 mm thickness, ε_r_ = 2.94, and 0.017 mm copper). The COTS are all assembled in a custom-printed circuit board (PCB) designed on the same substrate, also containing the phase shifters.

### 2.3. Receiver Calibration

The calibration technique is based on a four-step procedure implemented by switching two noise temperatures, *T_H_* and *T_C_*, as hot and cold temperatures, at both input ports, antenna and reference [31]. Subsequently, the output voltages are measured at each output and correction coefficients can be calculated. An updated procedure is presented that includes the detection stage and the calculation of the set of parameters from the measured output voltages. The conversion from microwave power to the voltage provided by the zero-biased square-law detectors are defined by voltage sensitivity, $\gamma_{DET}$, expressed as

(2) $$\begin{array}{c} \gamma_{DET}=\frac{V_{out}}{P_{DET}} \end{array}$$

where *V_out_* and *P_DET_* are the values for the DC output voltage and input available power at the detector, respectively. This parameter is considered in the set of variables resulting from the calibration process.

Calibration takes into account the non-ideal behavior of the subsystems that compose the receivers, such as leakages between branches, to correct them and perform a precise measurement [31]. The output voltages in terms of both input noise temperatures at the antenna and reference ports are expressed as

(3) $$\begin{array}{c} V_{k}=\alpha \cdot \left(A_{k}\cdot T_{ant}+R_{k}\cdot T_{ref}+N_{k}\cdot T_{rec}\right) \end{array}$$

with α as the conversion parameter in V/K. *A_k_*, *R_k_* and *N_k_* are constant values corresponding to each noise temperature at the antenna port, reference port and equivalent noise temperature of the receiver, respectively.

Subsequently, the output voltages, *V*_1_ to *V*_4_ in Figure 3, are measured by switching both input noise temperatures at each input port. The conversion parameter, α, is calculated from *V*_3_ as

(4) $$\begin{array}{c} \alpha =\frac{V_{3@\mathrm{TrefH}}-V_{3@\mathrm{TrefC}}}{T_{refH}-T_{refC}} \end{array}$$

where *T_refH_* and *T_refC_* are the hot and cold temperatures at the reference port, respectively. Then, the parameters are calculated as follows:

(5) $$\begin{array}{c} A_{1}=\bar{\alpha_{11}}=\frac{1}{\alpha }\cdot \frac{V_{1@\mathrm{TantH}}-V_{1@\mathrm{TantC}}}{T_{antH}-T_{antC}} \end{array}$$

(6) $$\begin{array}{c} A_{2}=\bar{\beta_{2}}=\frac{1}{\alpha }\cdot \frac{V_{3@\mathrm{TantH}}-V_{3@\mathrm{TantC}}}{T_{antH}-T_{antC}} \end{array}$$

(7) $$\begin{array}{c} A_{3}=\bar{\alpha_{3}}=\frac{1}{\alpha }\cdot \frac{V_{2@\mathrm{TantH}}-V_{2@\mathrm{TantC}}}{T_{antH}-T_{antC}} \end{array}$$

(8) $$\begin{array}{c} A_{4}=\bar{\alpha_{41}}=\frac{1}{\alpha }\cdot \frac{V_{4@\mathrm{TantH}}-V_{4@\mathrm{TantC}}}{T_{antH}-T_{antC}} \end{array}$$

(9) $$\begin{array}{c} R_{1}=\bar{\alpha_{12}}=\frac{1}{\alpha }\cdot \frac{V_{1@\mathrm{TrefH}}-V_{1@\mathrm{TrefC}}}{T_{refH}-T_{refC}} \end{array}$$

(10) $$\begin{array}{c} R_{3}=\bar{\beta_{3}}=\frac{1}{\alpha }\cdot \frac{V_{2@\mathrm{TrefH}}-V_{2@\mathrm{TrefC}}}{T_{refH}-T_{refC}} \end{array}$$

(11) $$\begin{array}{c} R_{4}=\bar{\alpha_{42}}=\frac{1}{\alpha }\cdot \frac{V_{4@\mathrm{TrefH}}-V_{4@\mathrm{TrefC}}}{T_{refH}-T_{refC}} \end{array}$$

(12) $$\begin{array}{c} N_{1}=2\cdot \bar{\alpha_{13}}=\frac{2}{\alpha }\cdot \frac{V_{1@T_{\mathrm{antC},\mathrm{refC}}}-\alpha \cdot A_{1}\cdot T_{\mathrm{antC}}-\alpha \cdot R_{1}\cdot T_{\mathrm{refC}}}{2\cdot T_{\mathrm{rec}}} \end{array}$$

(13) $$\begin{array}{c} N_{3}=\bar{\alpha_{3}}=\frac{1}{\alpha }\cdot \frac{V_{2@\mathrm{TantH}}-V_{2@\mathrm{TantC}}}{T_{antH}-T_{antC}} \end{array}$$

(14) $$\begin{array}{c} N_{4}=2\cdot \bar{\alpha_{43}}=\frac{2}{\alpha }\cdot \frac{V_{4@T_{\mathrm{antC},\mathrm{refC}}}-\alpha \cdot A_{4}\cdot T_{\mathrm{antC}}-\alpha \cdot R_{4}\cdot T_{\mathrm{refC}}}{2\cdot T_{\mathrm{rec}}} \end{array}$$

where *T_antH_* and *T_antC_* are the hot and cold temperatures, respectively, at the antenna port. In addition, the corrected equivalent receiver noise temperature, *T_rec_*, is calculated using the Y-factor as

(15) $$T_{rec}=\frac{\alpha \cdot \left(T_{refH}-Y\cdot T_{refC}\right)+\alpha \cdot A_{2}\cdot \left(1-Y\right)\cdot T_{antC}}{\alpha \cdot (Y-1)}$$

where *Y* = *V*_3__@ *TrefH*_/*V*_3__@ *TrefC*_ and *T_antC_* is the fixed temperature at the antenna port, while temperatures at the reference port are switched between hot and cold, *T_refH_* and *T_refC_*, respectively.

After the calibration parameters are calculated, temperature retrieval at the antenna port can be performed. Two noise temperatures at the reference port are employed to obtain the unknown temperature. Then, a switched noise source on its ON and OFF states, *T_refH_* and *T_refC_*, is used, and the following parameters are calculated to extract the unknown temperature:

(16) $$\begin{array}{c} \alpha_{\mathrm{med}}=\frac{{\left(V_{1}+V_{4}\right)}_{@T_{\mathrm{refH}}}-{\left(V_{1}+V_{4}\right)}_{@T_{\mathrm{refC}}}}{\left(T_{\mathrm{refH}}-T_{\mathrm{refC}}\right)\cdot \left(R_{1}+R_{4}\right)} \end{array}$$

(17) $$T_{\mathrm{recmed}}=\frac{\left(T_{\mathrm{refH}}-Y_{\mathrm{med}}\cdot T_{\mathrm{refC}}\right)}{\left(Y_{\mathrm{med}}-1\right)}$$

where *Y_med_* = *V*_3_ *@ T_refH_*/*V*_3_ *@ T_refC_*. Finally, the measured value of the unknown antenna temperature, *T_antmed_*, is given by

(18) $$T_{\mathrm{antmed}}=\frac{\left(\frac{V_{2@T_{\mathrm{refC}}}}{\alpha_{\mathrm{med}}}-R_{3}\cdot T_{\mathrm{refC}}-N_{3}\cdot T_{\mathrm{recmed}}\right)}{A_{3}}\cdot \left(1+A_{2}\right).$$

## 3. Results

This section describes the experimental results of the multifrequency radiometers, as well as their conversion performances. The characterization of each receiver, depicted in Figure 3, can be divided into two steps: the microwave part of the receivers from the input ports, labeled (A) and (R) in Figure 3, to ports (1) to (4) prior to detection stage, and then, the conversion from microwave power to detected voltages with the detectors added to the receiver. In addition, two noise sources are also characterized, which are employed for calibrating and measuring the radiometers. The characterization of the radiometers is completed by performing their calibration and, subsequently, temperature retrieval with a set of unknown sources.

### 3.1. Microwave Chain

The SMA connectorized prototypes are characterized in terms of scattering parameters and noise temperatures. A precision network analyzer (PNA) E8364A and a noise figure analyzer N8975A with a noise source N4000A, all of them from Keysight Technologies, are used for each measurement, respectively.

**Figure 5:**
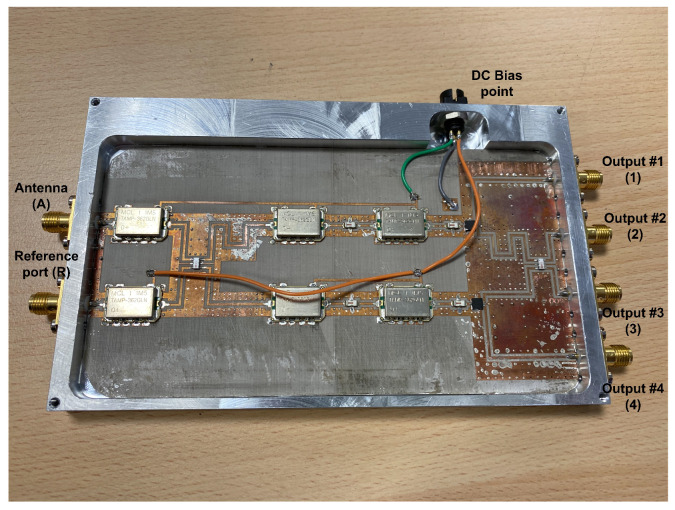
Representative assembly of the radiometers in a chassis, provided with SMA connectors (size 146.6 × 90.2 × 11 mm).

**Figure 6:**
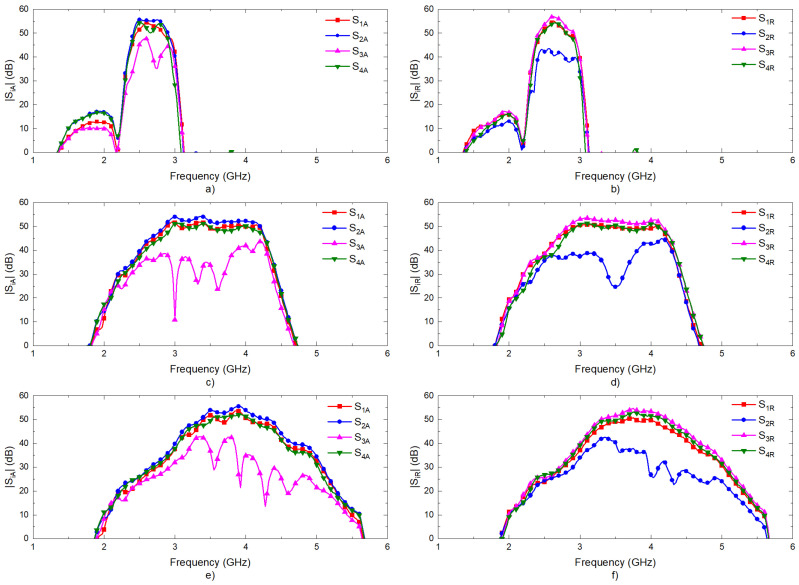
Measurements of the transmission coefficients of the three radiometers. Transmissions from antenna port (A) to other outputs: (**a**) band B1; (**c**) band B2; (**e**) band B3. Transmissions from reference port (R) to other outputs: (**b**) band B1; (**d**) band B2; (**f**) band B3.

**Figure 7:**
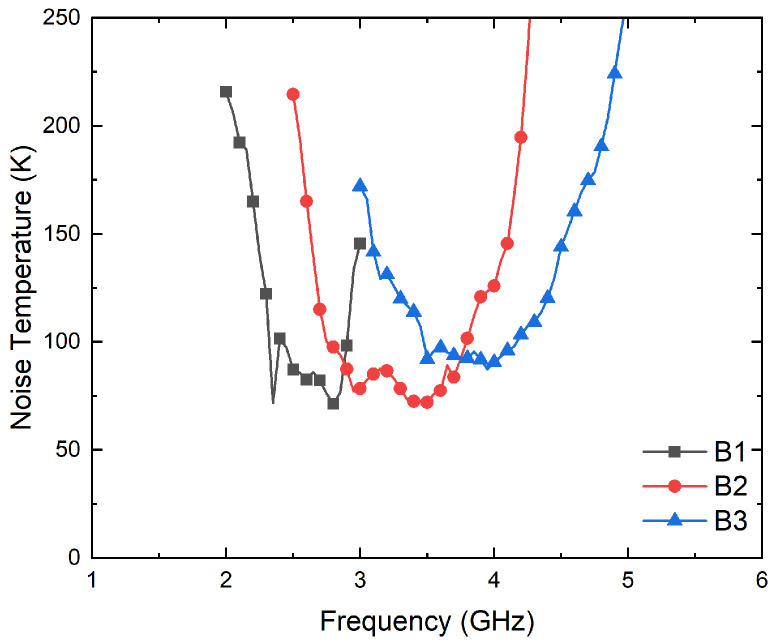
Noise temperature measurements of the three radiometers from the antenna port (A) to output (2).

The transmission coefficients are measured for the set of radiometers from each input port, labeled as antenna (A) and reference (R) in Figure 3, to each one of the output ports, labeled from (1) to (4). A representative assembly of the radiometers is shown in Figure 5, and the results are shown in Figure 6. Maximum values of the transmissions are obtained for the output labeled as (2) in Figure 3 for the antenna input, whereas output (3) corresponds to the reference. Average gain values of 54.1, 52.2 and 51.3 dB are measured within the B1, B2 and B3 bands, respectively, for the direct antenna transmission path (S_2A_), whereas 53.7, 51.9 and 50.9 dB are measured for the reference transmission path (S_3R_). Isolation values better than 19 dB are obtained. The noise temperatures of the three receivers are shown in Figure 7. Average noise temperatures of 89, 90.6 and 100.2 K for B1, B2 and B3 receivers are provided around their center frequencies, 2.7, 3.5 and 4.1 GHz, respectively. The effective bandwidths [48,49,50] of the radiometers are 0.51, 1.49 and 1.13 GHz for B1, B2 and B3 bands, respectively.

### 3.2. Diode Detector

Four units of the detector model SMD0112 from Fairview Microwave [47] are measured. The zero-bias detectors with negative voltage conversion are configured to operate following a square-law response for an input power dynamic range, providing an output voltage directly proportional to the power of the input signal.

**Figure 8:**
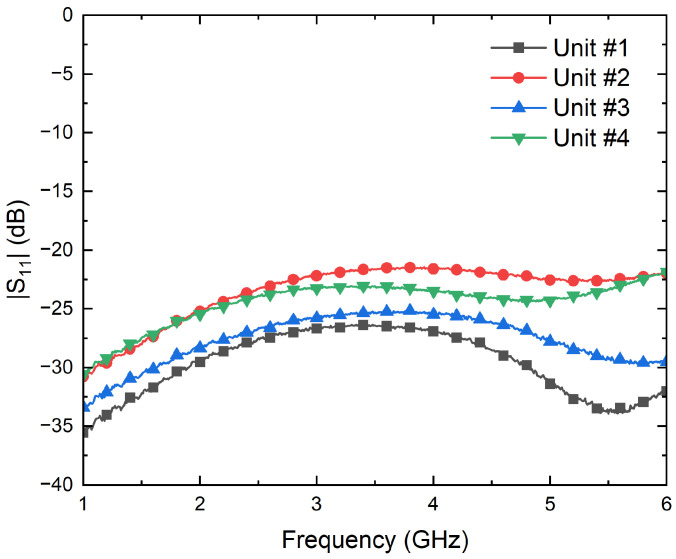
Measurement of the input reflection coefficients of the four units of the SMD0112 detectors.

**Figure 9:**
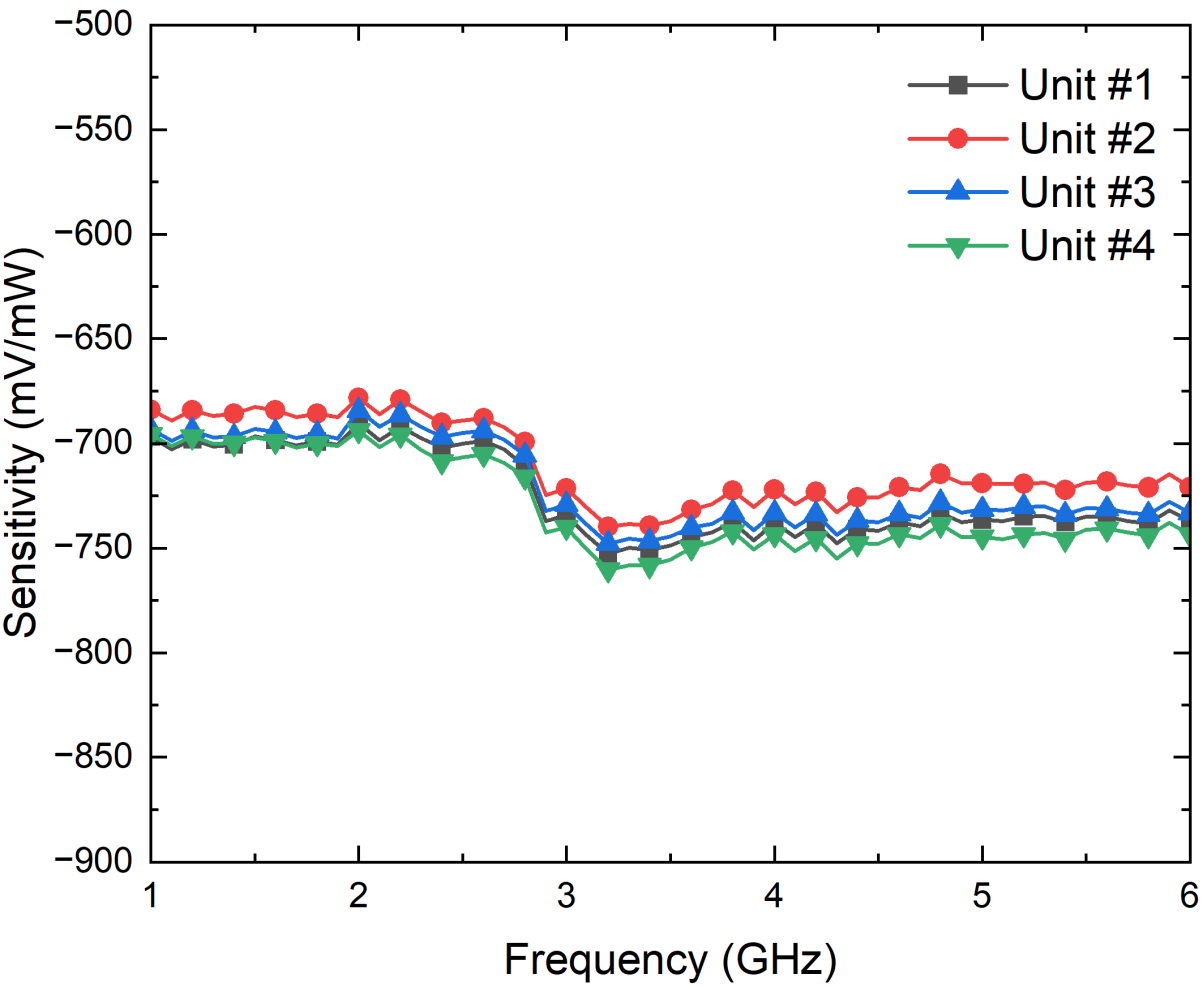
Measurement of the sensitivities, in mV/mW, of the four units of the SMD0112 detectors for an input power of −25 dBm.

First, their input reflection coefficients are characterized using the PNA. Then, the conversion from microwave power to DC voltages is measured to calculate their voltage sensitivities, $\gamma_{DET}$, by means of a signal generator E83650B and a multimeter 34401A, both from Keysight Technologies. The results are shown in Figure 8 and Figure 9 in terms of the input reflection coefficient and sensitivity, respectively. The four units exhibit an input matching better than −20 dB in the band of interest, whereas average sensitivities of −725 mV/mW are measured for input powers of −25 dBm. In addition, the four units show a measured 1-dB compression point of around −12.5 dBm.

### 3.3. NC520 Noise Source

Two units of the noise source model NC520 from Noisecom [51] are individually characterized. Both units are involved afterward in the calibration process, as well as in the real-time measurement of an unknown source.

The NC520 is a switchable noise source that provides two power levels corresponding to its ON or OFF states by appropriate selecting of the control voltage level V_TTL_ [51]. The OFF state approximately corresponds to an equivalent noise temperature of 300 K when a DC voltage supply of V_CC_ = 5 V and a control voltage V_TTL_ = 5 V are applied, with a negligible current consumption. On the other hand, when V_TTL_ is switched to 0 V, the ON state is activated and measured to calculate its equivalent noise temperature. These two power levels provided by each noise source state are required to perform the calibration and retrieve the unknown temperature at antenna input, as described in previous sections. Since the theoretical value of the excess noise ratio of a NC520 is 25 dB [51], attenuators are connected at their outputs to avoid saturation of the receivers. In addition, the use of attenuators improves the noise source reflection coefficient and reduces the uncertainty of the measurement since the change in the reflection coefficient between ON and OFF states of the noise source is mitigated.

**Figure 10:**
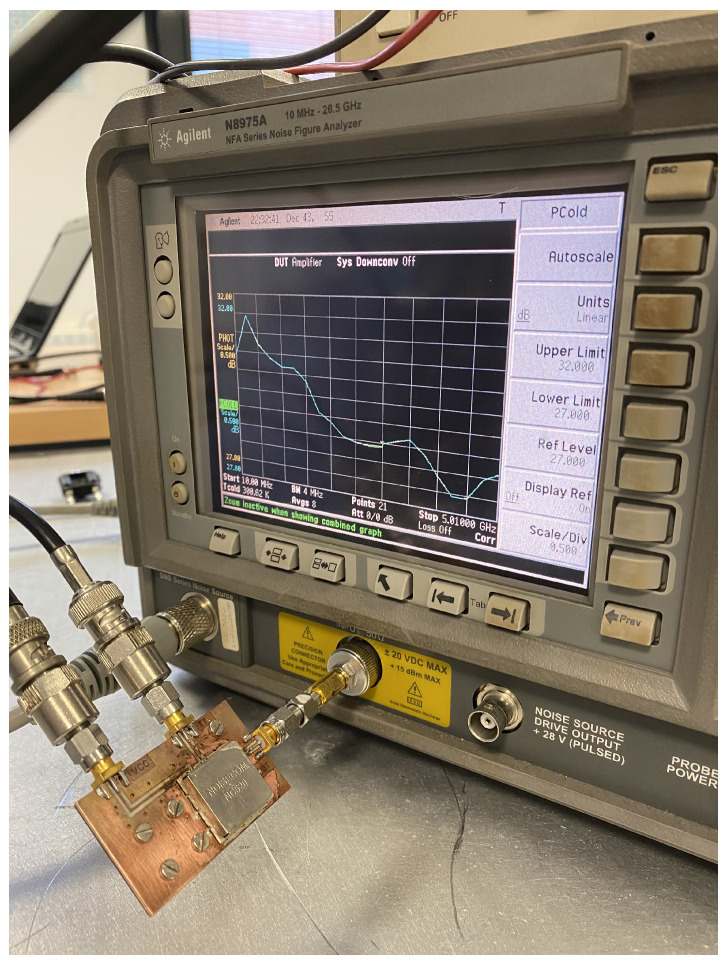
Noise characterization of one of the noise sources of NC520.

**Figure 11:**
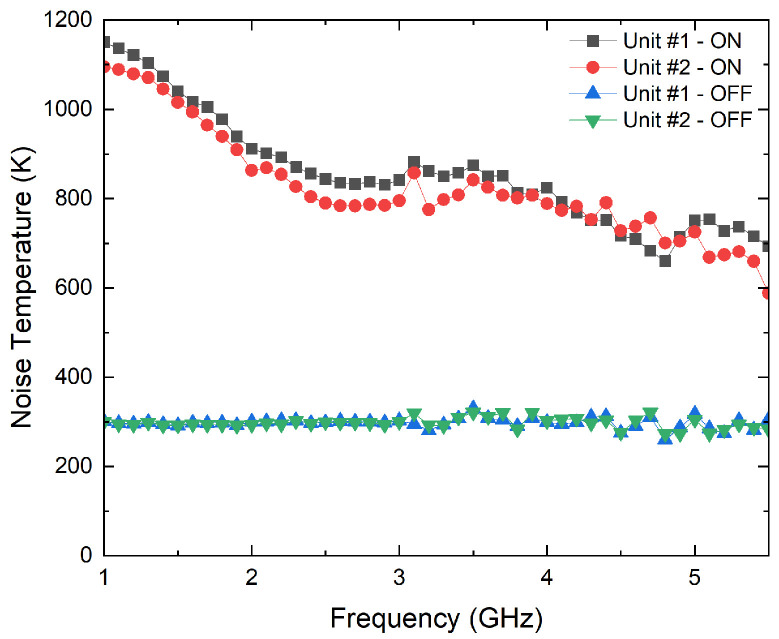
Noise characterization of both noise sources of NC520 attenuated 26 dB.

A noise figure analyzer N8975A is employed for the characterization of the ON/OFF states of the noise sources. The analyzer is calibrated using a N4000A noise source, and then, each NC520 is connected to the analyzer. Initially, the NC520 sources are individually measured, and they provide an excess noise radio of around 28 dB on their ON states. Thus a 26 dB attenuator at their outputs is used to reduce the input noise power into the receivers. The measurement process is shown in Figure 10, and the results for both sources, in terms of the noise temperatures provided in each state, in Figure 11. A current consumption of less than 8 mA is measured in the ON state. Noise temperatures on the order of 800 K are provided for both sources within B1, B2 and B3 bands of the radiometers on their ON states, whereas around 300 K is measured on their OFF states.

### 3.4. Calibration Parameters Extraction

The calibration procedure is applied to the receivers by means of the two units of the NC520 noise source attenuated 26 dB. They are switched to swap their states at both input ports.

Two single equivalent noise temperature values are required for each noise source used in the calibration procedure. Since the noise power provided by the noise sources on their ON states is not flat over the frequency range, the equivalent noise temperature is calculated by integrating the noise source response over the frequency band of each radiometer, taking into account the transmission coefficients of the direct branch in which each noise source is connected. The NC520 unit #1 is connected to the antenna port, labeled as (A) in Figure 3, and its direct output corresponds to port (2). On the other hand, unit #2 is integrated between the reference load port, labeled as (R), and output (3) in Figure 3. Thus, the equivalent noise temperature for each state of the noise sources, *T_eq_*, is calculated as

(19) $$T_{\mathrm{eq}}=\frac{\sum_{f_{1}}^{f_{2}}\left(T_{NS}\left(f\right)\cdot G\left(f\right)\right)}{\sum_{f_{1}}^{f_{2}}G\left(f\right)}$$

where *T_NS_*(*f*) is the measured noise temperature provided by each noise source on the ON state, and *G*(*f*) is the power gain of the corresponding transmission path as *|S_*2*A_*|^2^ or *|S_*3*R_*|^2^. Finally, *f*_1_ and *f*_2_ are the initial and final measured frequencies shown in Figure 11. The noise sources on their OFF states show a flat response, so it is considered that they provide a constant value, obtained as the average value within the bandwidth. The values of the equivalent noise temperatures of both units on their ON and OFF states are listed in Table 3.

**Figure 12:**
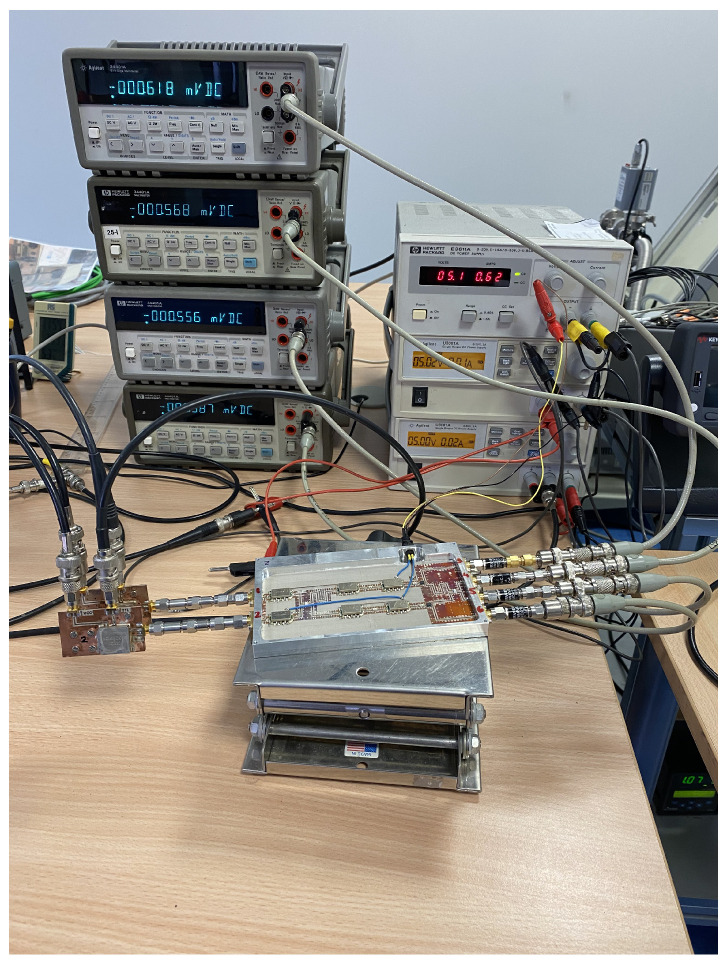
Setup for the calibration measurement of one of the radiometers using both NC520 noise sources to extract the calibration parameters.

Thus, the corresponding pair of values of the noise sources units #1 and #2 are employed as *T_antH_*/*T_antC_* and *T_refH_*/*T_refC_*, respectively, for the extraction of the parameters depending on the frequency band of the receiver and the state of the source. The output voltages are measured with four multimeters, 34401A, and the calibration parameters are calculated and listed in Table 4, Table 5 and Table 6 for each one of the three receivers, respectively. The listed values are normalized to α for each frequency band, which is also defined in the table captions. The measurement process of one of the receivers is shown in Figure 12.

### 3.5. Temperature Retrieval

Experimental tests are performed to validate the method to retrieve the unknown temperatures provided by objects. Several sources are connected to the antenna port mimicking an unknown temperature, and the output voltages are measured by switching the noise source connected to the reference port. Initially, a 50 Ω load and a noise source are employed as inputs. Finally, a test using an antenna is performed to demonstrate the feasibility of the method when directly matched to biological tissues. The following subsections describe the measurements and the temperature retrieval for each case. The NC520 noise source attenuated 26 dB is employed as a switching noise source for all configurations.

#### 3.5.1. 50 Ω Load

**Figure 13:**
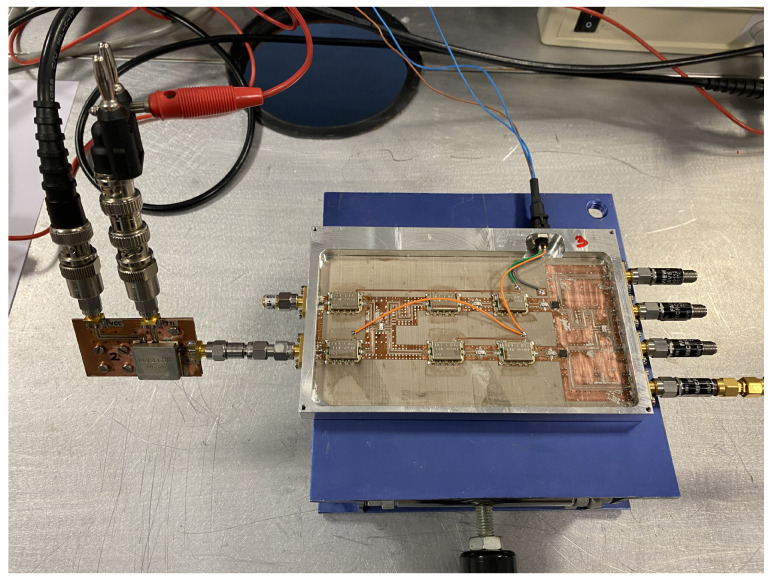
Temperature retrieval using a 50 Ω load as input at the antenna port.

A 50 Ω load is connected to the antenna port to retrieve its equivalent temperature, as shown in Figure 13. The measured voltages for each radiometer are listed in Table 7. Table 8 lists the temperatures calculated using the equations from (16)–(18), and the values are compared with an external measurement using an infrared thermometer pointing at the rear face of the load. A great consistency between the provided values by the radiometer and external measurements is observed.

#### 3.5.2. Noise Source 346C

**Figure 14:**
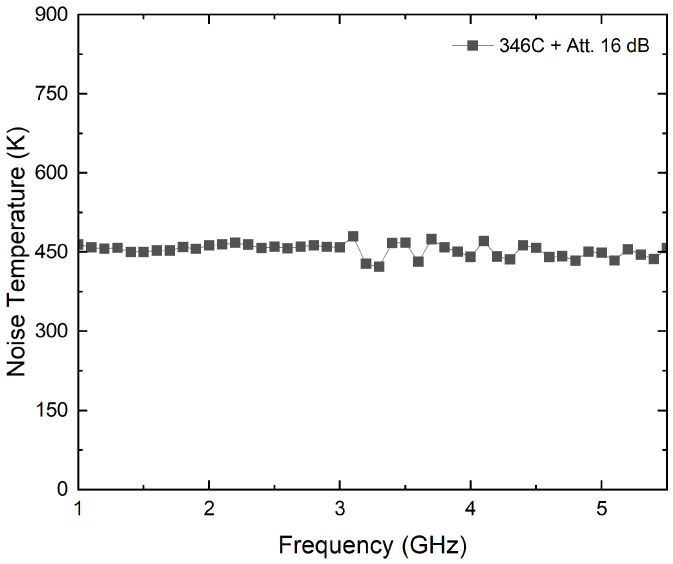
Noise characterization of noise source 346C attenuated to 16 dB.

**Figure 15:**
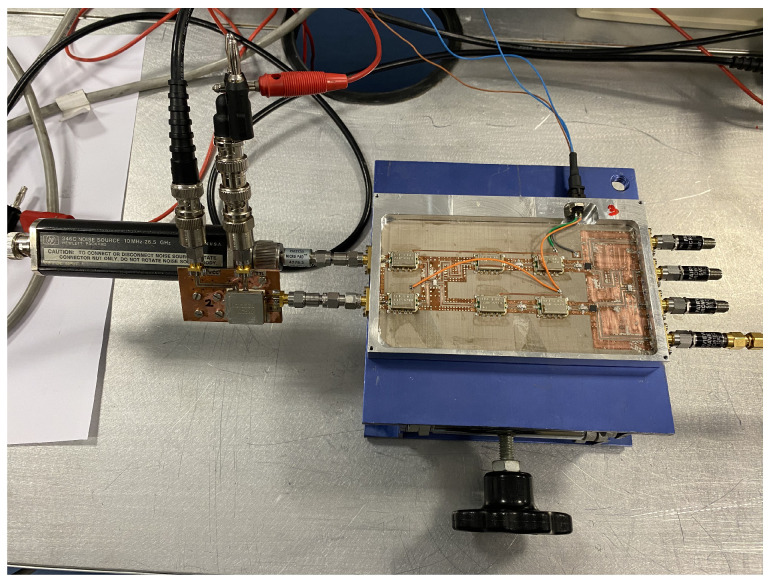
Temperature retrieval using a noise source 346C attenuated to 16 dB as input at the antenna port.

A noise source model, 346C, from Keysight Technologies, with a theoretical excess noise ratio of 16 dB, is employed to validate the method. To avoid receivers’ saturation, 16 dB attenuation is connected to the output of the noise source. The attenuated noise source shows a flat noise temperature over the frequency, as depicted in Figure 14. Thus, a constant noise temperature is considered for all the bands, with 453 K as the average value. Figure 15 shows the assembly for this experimental test.

The measurement procedure is applied, and the voltages for each radiometer are listed in Table 9, while the temperatures are calculated in Table 10. The values provided show a deviation lower than 0.1% from the measured noise temperature of the noise source.

#### 3.5.3. Test over Biological Tissues

**Figure 16:**
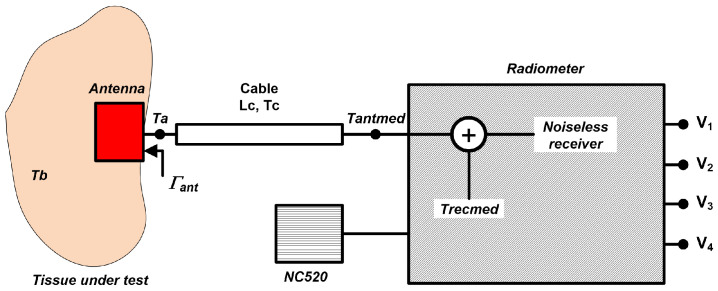
Simplified schematic for retrieving the temperature of a tissue, *T_b_*, when an antenna is connected to the radiometers.

Finally, a test of biological tissues is performed by connecting a probe antenna to the radiometer’s input. The calculated temperatures, *T_antmed_*, at radiometer inputs should be translated to the plane of the antenna *T_a_*. The schematic shown in Figure 16 depicts the connection of the involved components and the temperatures at each point and for each component. Therefore, the losses of the cable that connects the antenna to the radiometer input and the reflection coefficient of the probe antenna should be corrected to calculate the temperature of the tissue, *T_b_*.

Thus, *T_a_* is given by

(20) $$\begin{array}{c} T_{a}=T_{b}\cdot (1-|\Gamma_{\mathrm{ant}}{|}^{2})+T_{r}\cdot {\left|\Gamma_{\mathrm{ant}}\right|}^{2} \end{array}$$

where *T_b_* is the temperature of the tissue, Γ_ant_ is the average reflection coefficient of the antenna over the effective bandwidth of each radiometer band and *T_r_* is the noise temperature of the radiometer and cable at the antenna plane, calculated as

(21) $$\begin{array}{c} T_{r}=\frac{T_{\mathrm{recmed}}+T_{c}\cdot \left(10^{\frac{L_{c}}{10}}-1\right)}{10^{\frac{L_{c}}{10}}} \end{array}$$

where *T_recmed_* is the equivalent noise temperature of the receiver provided by the method, and *T_c_* and *L_c_* are the temperature and losses in dB of the cable, respectively. The losses in the cable are individually measured and approximated, within the frequency range of the radiometers, by

(22) $$\begin{array}{c} L_{c}\left(dB\right)=-0.059-0.043\cdot x+0.001\cdot x^{2} \end{array}$$

where *x* is the frequency in GHz and corresponds to the center frequency of each receiver band (B1, B2 or B3). Then, *T_a_* is obtained at the input plane of the radiometer correcting for the losses in the cable and *T_b_* is calculated as

(23) $$\begin{array}{c} T_{b}=\frac{T_{\mathrm{antmed}}\cdot 10^{\frac{L_{c}}{10}}-\left(T_{recmed}+T_{c}\cdot \left(10^{\frac{L_{c}}{10}}-1\right)\right)\cdot \frac{|\Gamma_{\mathrm{ant}}{|}^{2}}{10^{\frac{L_{c}}{10}}}-T_{c}\cdot \left(10^{\frac{L_{c}}{10}}-1\right)}{\left(1-|\Gamma_{\mathrm{ant}}{|}^{2}\right)} \end{array}$$

where *T_antmed_* is the measurement.

**Figure 17:**
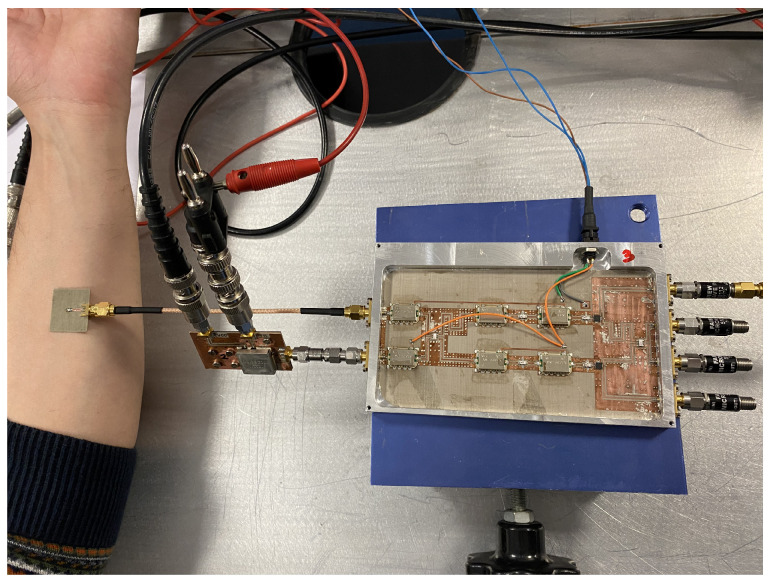
Temperature retrieval of biological tissues by means of an antenna matched to the body (forearm).

An asymmetric, double-crossed H-shaped slot antenna [52,53] is used to retrieve the temperature from body tissues. The measurement with the antenna over the forearm is shown in Figure 17. The output voltages for each receiver are listed in Table 11, whereas Table 12 registers the temperature retrieval. To validate the figures obtained, a measurement of the skin surface using an infrared thermometer is performed. A skin temperature of 307.8 K is measured, which only corresponds to the skin temperature, and it is expected to differ from the retrieved value.

These tests using the multifrequency receiver demonstrate that the system is sensitive to detect temperature variations within biological tissues, and they are able to retrieve unknown temperatures from any source. Yet, the analysis of the depth reached depending on the operation frequency is still pending to precisely know the targetted point inside the tissues.

## 4. Discussion

A pseudo-correlation configuration is proposed, providing a reduction in the noise temperature of the receiver, which is significant for measuring tiny power levels. A reduction in the noise temperature of more than 40 K in the 3.5-GHz band is observed compared to other works in the same band, and the proposed radiometers also show better noise response at lower frequency bands [31]. Furthermore, a method using a single calibration improves the observation time compared to Dicke proposals without the need to periodically switch to a reference load.

The tests described in this work are performed at the ambient temperature of the laboratory. However, thermal stability is required to test the systems under the same ambient conditions. Ideally, a laboratory with a controlled environment in terms of temperature and humidity is needed to experimentally test the performance to avoid drifts in devices’ response, such as noise sources or radiometers, due to temperature changes.

The work presented stands as the proof-of-concept of the proposed methodology and its validation by measurements over biological tissues. Nevertheless, further analysis is required when different body areas or different individual’s measurements are considered since the reflection coefficient, Γ_ant_, depends on the stack of tissues under investigation.

Once the proper performance of the proposed calibration and measurement procedure of temperature retrieval is demonstrated, the forthcoming approaches are focused on penetration depth measurements. As a multifrequency system, different depths are expected for each working frequency, and they should be demonstrated. For this purpose, experimental tests require materials mimicking biological tissues. Thus, phantoms are going to be employed, replicating dielectric properties in terms of relative permittivity and conductivity and the structure, if required for anthropomorphic models, of the biological tissues intended. In addition, long-term stability and extended shelf-life are required to maintain phantoms’ characteristics over time. Previous attempts employed phantoms based on polyvinyl alcohol cryogel as a gelling agent, using well-known models [54,55]. In addition, these phantoms enable multimodality operation, combining microwave and ultrasound [56] techniques facilitating the guidance of microwave measurement.

Further research is required to extract 3D profiles of internal temperatures [23]. In-depth measurements using phantoms together with the multifrequency radiometers enable the development of 3D profile models depending on frequency, predicting temperature distribution and distance in which anomalous temperatures can be detected.

In addition, new technological advances for MWR systems are required to improve accuracy. In this context, new designs for near-field probes are necessary to improve the matching over biological tissues, maximizing the provided power to the radiometers. Approaches for microwave antennas have been under research over the last years, including configurations using spiral resonators [57], a combination of a symmetric dipole and an annular frame [58], a meander patch antenna [59] or a dielectric-filled waveguide solution [60], among others.

## 5. Conclusions

This paper presents a new proposal for retrieving the temperature of objects or biological tissues by means of pseudo-correlation radiometers. The performances of multifrequency radiometers are assessed, showing significant results in retrieving unknown temperatures. The calibration and characterization method is demonstrated by using a single calibration and a set of outputs to recalibrate the radiometers’ performances. The receivers’ topology is intended to measure continuously, preventing users’ discomfort and cyclical calibration but enabling signs of drifts in receivers’ responses by use of a set of outputs. The radiometers show high sensitivity with a simultaneous low-noise response, lower than 100 K in all bands. Temperature retrievals are performed using noise sources to validate the procedure, achieving errors lower than 0.1% with respect to the temperature provided by the noise sources. Furthermore, a near-field measurement from the human body is also presented, with temperature calculations of around 310 K. The results from this work demonstrate that a feasible temperature retrieval is provided using the proposed method with significant results.

## Figures and Tables

**Table 1:** Definition of frequency bands for the set of radiometers.

Band	Center Frequency
B1	2.7 GHz
B2	3.5 GHz
B3	4.1 GHz

**Table 2:** COTS components employed in the receiver design for each frequency band.

Device	Part Number	Frequency Band
Low-noise Amplifier (LNA)	TAMP-362GLN+ (MiniCircuits) [39]	B1, B2, B3
90° Hybrid Coupler	QCN-27+ (MiniCircuits) [40] QCN-45+ (MiniCircuits) [41]	B1 B2, B3
Band-pass Filter (BPF)	BFCN-2700+ (MiniCircuits) [42] BFCN-3500+ (MiniCircuits) [43] BFCN-4440+ (MiniCircuits) [44]	B1 B2 B3
Power Splitter	GP2Y+ (MiniCircuits) [45] GP2X+ (MiniCircuits) [46]	B1 B2, B3
Schottky diode detector	SMD0112 (Fairview Microwave) [47]	B1, B2, B3

**Table 3:** Equivalent noise temperatures of both NC520 units attenuated 26 dB for the three frequency bands on their ON and OFF states.

Frequency Band	Equivalent Noise Temperature			
	Unit #1-ON	Unit #1-OFF	Unit #2-ON	Unit #2-OFF
B1	838 K		786 K	
B2	839 K	303 K	803 K	303 K
B3	821 K		798 K	

**Table 4:** Calibration parameters calculated for the B1 band receiver, centered at 2.7 GHz, normalized to α = −1.261 × 10^−6^ V/K.

Parameter	Output			
	k = 1	k = 2	k = 3	k = 4
*A_k_*	$\bar{\alpha_{11}}$ = 0.612	$\bar{\beta_{2}}$ = 0.089	$\bar{\alpha_{3}}$ = 1.082	$\bar{\alpha_{41}}$ = 0.633
*R_k_*	$\bar{\alpha_{12}}$ = 0.617	1	$\bar{\beta_{3}}$ = 0.056	$\bar{\alpha_{42}}$ = 0.563
*N_k_*	2 · $\bar{\alpha_{13}}$ = 1.002	1	$\bar{\alpha_{3}}$ = 1.082	2 · $\bar{\alpha_{43}}$ = 0.992

**Table 5:** Calibration parameters calculated for the B2 band receiver, centered at 3.5 GHz, normalized to α = −1.990 × 10^−6^ V/K.

Parameter	Output			
	k = 1	k = 2	k = 3	k = 4
*A_k_*	$\bar{\alpha_{11}}$ = 0.671	$\bar{\beta_{2}}$ = 0.047	$\bar{\alpha_{3}}$ = 1.102	$\bar{\alpha_{41}}$ = 0.567
*R_k_*	$\bar{\alpha_{12}}$ = 0.607	1	$\bar{\beta_{3}}$ = 0.057	$\bar{\alpha_{42}}$ = 0.614
*N_k_*	2 · $\bar{\alpha_{13}}$ = 1.144	1	$\bar{\alpha_{3}}$ = 1.102	2 · $\bar{\alpha_{43}}$ = 1.012

**Table 6:** Calibration parameters calculated for the B3 band receiver, centered at 4.1 GHz, normalized to α = −1.904 × 10^−6^ V/K.

Parameter	Output			
	k = 1	k = 2	k = 3	k = 4
*A_k_*	$\bar{\alpha_{11}}$ = 0.604	$\bar{\beta_{2}}$ = 0.047	$\bar{\alpha_{3}}$ = 1.066	$\bar{\alpha_{41}}$ = 0.572
*R_k_*	$\bar{\alpha_{12}}$ = 0.445	1	$\bar{\beta_{3}}$ = 0.042	$\bar{\alpha_{42}}$ = 0.701
*N_k_*	2 · $\bar{\alpha_{13}}$ = 1.009	1	$\bar{\alpha_{3}}$ = 1.066	2 · $\bar{\alpha_{43}}$ = 1.242

**Table 7:** Output voltages for the 50 Ω load at the antenna port.

Band	Antenna Port	Reference Port	V_1_ (mV)	V_2_ (mV)	V_3_ (mV)	V_4_ (mV)
B1	50 Ω	NC520 ON NC520 OFF	−0.955 −0.593	−0.587 −0.550	−1.137 −0.532	−0.909 −0.575
B2	50 Ω	NC520 ON NC520 OFF	−1.673 −1.061	−1.033 −0.964	−1.874 −0.870	−1.583 −0.969
B3	50 Ω	NC520 ON NC520 OFF	−1.235 −0.809	−0.921 −0.877	−1.740 −0.809	−1.648 −0.989

**Table 8:** Temperature retrieval for the 50 Ω load.

Band	Temperature Calculation (K)	Temperature Provided by an Infrared Thermometer (K)
B1	303.87	304.1
B2	303.36	303.5
B3	304.65	304.8

**Table 9:** Output voltages for the noise source 346C attenuated to 16 dB at the antenna port.

Band	Antenna Port	Reference Port	V_1_ (mV)	V_2_ (mV)	V_3_ (mV)	V_4_ (mV)
B1	346C + Att. 16 dB	NC520 ON NC520 OFF	−1.030 −0.683	−0.753 −0.726	−1.112 −0.535	−0.985 −0.668
B2	346C + Att. 16 dB	NC520 ON NC520 OFF	−1.851 −1.241	−1.338 −1.280	−1.903 −0.885	−1.738 −1.125
B3	346C + Att. 16 dB	NC520 ON NC520 OFF	−1.393 −0.986	−1.216 −1.148	−1.725 −0.813	−1.784 −1.143

**Table 10:** Temperature retrieval for the noise source 346C attenuated to 16 dB.

Band	Temperature Calculation (K)
B1	452.64
B2	452.93
B3	452.87

**Table 11:** Output voltages for an antenna matched to biological tissues (forearm) at the antenna port.

Band	Antenna Port	Reference Port	V_1_ (mV)	V_2_ (mV)	V_3_ (mV)	V_4_ (mV)
B1	Forearm	NC520 ON	−0.952	−0.580	−1.086	−0.873
		NC520 OFF	−0.571	−0.537	−0.518	−0.561
B2	Forearm	NC520 ON	−1.640	−1.050	−1.867	−1.570
		NC520 OFF	−1.030	−0.934	−0.864	−0.970
B3	Forearm	NC520 ON	−1.226	−0.909	−1.681	−1.602
		NC520 OFF	−0.817	−0.870	−0.799	−0.972

**Table 12:** Temperature retrieval for the forearm.

Band	Temperature Calculation (K)
B1	309.93
B2	309.75
B3	309.43

## Data Availability

Not applicable.
